# Characterization of transcriptome changes in saline stress adaptation on *Leuciscus merzbacheri* using PacBio Iso-Seq and RNA-Seq

**DOI:** 10.1093/dnares/dsae019

**Published:** 2024-05-29

**Authors:** Dan Yu, Min Zhou, Wenjun Chen, Zufa Ding, Cheng Wang, Yuting Qian, Yang Liu, Shunping He, Liandong Yang

**Affiliations:** School of Ecology and Environment, Tibet University, Lhasa, 850000, China; State Key Laboratory of Freshwater Ecology and Biotechnology, Institute of Hydrobiology, Chinese Academy of Sciences, Wuhan, 430072, China; School of Life Sciences, Jianghan Universily, Wuhan 430056, China; State Key Laboratory of Freshwater Ecology and Biotechnology, Institute of Hydrobiology, Chinese Academy of Sciences, Wuhan, 430072, China; College of Advanced Agricultural Sciences, University of Chinese Academy of Sciences, Beijing 100049, China; State Key Laboratory of Freshwater Ecology and Biotechnology, Institute of Hydrobiology, Chinese Academy of Sciences, Wuhan, 430072, China; College of Fisheries, Huazhong Agricultural University, Wuhan, 430070, China; State Key Laboratory of Freshwater Ecology and Biotechnology, Institute of Hydrobiology, Chinese Academy of Sciences, Wuhan, 430072, China; College of Advanced Agricultural Sciences, University of Chinese Academy of Sciences, Beijing 100049, China; State Key Laboratory of Freshwater Ecology and Biotechnology, Institute of Hydrobiology, Chinese Academy of Sciences, Wuhan, 430072, China; College of Advanced Agricultural Sciences, University of Chinese Academy of Sciences, Beijing 100049, China; State Key Laboratory of Freshwater Ecology and Biotechnology, Institute of Hydrobiology, Chinese Academy of Sciences, Wuhan, 430072, China; College of Advanced Agricultural Sciences, University of Chinese Academy of Sciences, Beijing 100049, China; State Key Laboratory of Freshwater Ecology and Biotechnology, Institute of Hydrobiology, Chinese Academy of Sciences, Wuhan, 430072, China; State Key Laboratory of Freshwater Ecology and Biotechnology, Institute of Hydrobiology, Chinese Academy of Sciences, Wuhan, 430072, China

**Keywords:** *L. merzbacheri*, PacBio Iso-Seq, RNA-seq, saline water, adaptation

## Abstract

*Leuciscus merzbacheri* is a native fish species found exclusively in the Junggar Basin in Xinjiang. It exhibits remarkable adaptability, thriving in varying water conditions such as the saline waters, the semi-saline water, and the freshwater. Despite its significant economic and ecological value, the underlying mechanisms of its remarkable salinity tolerance remain elusive. Our study marks the first time the full-length transcriptome of *L. merzbacheri* has been reported, utilizing RNA-Seq and PacBio Iso-Seq technologies. We found that the average length of the full-length transcriptome is 1,780 bp, with an N50 length of 2,358 bp. We collected RNA-Seq data from gill, liver, and kidney tissues of *L. merzbacheri* from both saline water and freshwater environments and conducted comparative analyses across these tissues. Further analysis revealed significant enrichment in several key functional gene categories and signalling pathways related to stress response and environmental adaptation. The findings provide a valuable genetic resource for further investigation into saline-responsive candidate genes, which will deepen our understanding of teleost adaptation to extreme environmental stress. This knowledge is crucial for the future breeding and conservation of native fish species.

## 1. Introduction

*Leuciscus merzbacheri*, commonly known as the Xinjiang Yarrowfish, is a member of the Cyprinidae family.^1^ This fish is unique to the Junggar Basin’s water systems in Xinjiang, making it an endemic species to this region.^2^ It has high edible value and is the main economic fish in this area, with broad breeding prospects.^1^ In recent years, the fish population has experienced a notable decline as a result of the impact of human economic activities. This decline was so severe that in 1998, *L. merzbacheri* was listed as an endangered species on China’s Red List.^3^ Remarkably, *L. merzbacheri* shows a strong ability to adapt and survive in various water conditions, including saline waters like those found at the Jinghe River estuary leading into Ebinur Lake, semi-saline waters of Sayram Lake, and freshwater environments like the Boltara River.^4^ Ebinur Lake, characterized by its high sulfate salt concentration, is a significant saline lake in the Junggar Basin. As the second-largest and the largest saline lake in Xinjiang, it plays a crucial role in maintaining the ecological balance of the region and all of Northern Xinjiang. The lake is also a vital habitat and migratory stopover for birds in Xinjiang.^5^ Since the 1950s, the lake has been shrinking due to evaporation exceeding both precipitation and inflows, leading to an increase in salinity. This change in the lake’s environment means that no fish, except for those in the estuary areas like *L. merzbacheri*, can now survive in it. Geological and biological evidence suggests that the freshwater populations of *L. merzbacheri* in Ebinur Lake have rapidly evolved over the past several million years, developing a tolerance to high salinity. This adaptation is a testament to the species’ remarkable resilience in the face of environmental changes.

*L. merzbacheri* holds significant economic value for the local communities around Ebinur Lake and plays a crucial ecological role as a primary food source for wild birds migrating south. Despite its importance, the secrets behind its remarkable ability to withstand high salinity levels remain largely unknown. Past research on *L. merzbacheri* primarily focussed on its distribution, diet, and reproduction, with only a handful of studies delving into its physiology and genetics.^6,7^ To date, only a limited number of genetic markers have been identified for population genetics and phylogenetic analysis.^8,9^ The scientific community has increasingly acknowledged the importance of *L. merzbacheri*, recognizing its potential in aquaculture, especially within the saline lakes of Northern China. This interest has spurred breeding programs to gain a deeper understanding of the fish’s physiological and genetic adaptations to saline environments. The advent of high-throughput sequencing technology has recently opened new avenues for exploring the mechanisms of saline tolerance in *L. merzbacheri*, offering hope for more comprehensive insights into this resilient species.^10^

Comparative studies of organisms living in different environments can shed light on how species respond to environmental variations. Sometimes, scientists employ manual methods in experiments to create varying conditions for comparison.^11^ For a deeper understanding of the physiological and genetic adaptations of *L. merzbacheri* to saline environments, comparing populations from saline and freshwater habitats is a practical approach. Previous research, such as the transcriptome sequencing studies on *Leuciscus waleckii* in the highly alkaline Dali Nor Lake, has explored molecular adaptation mechanisms in extreme environments.^12^ Fortunately, freshwater populations of *L. merzbacheri* have been discovered in the Boltara River Aheqi Farm. *L. merzbacheri* annually through anadromous spawning migration, with entering freshwater rivers for breeding during the reproductive season. Both populations of *L. merzbacheri* stem from the same ancestors and maintain a consistent genetic background. These fish offer distinctive natural samples for investigating gene expression changes in high-saline environments.^13^

The transcriptome, representing all genes expressed in a cell or group of cells, is a valuable resource for investigating gene expression and evolutionary processes.^14^ Over the past decade, RNA-Seq has been extensively used for differential gene expression analysis in various osteichthyans under different environmental challenges.^15,16^ Although many models and non-model organisms’ transcriptomes have been generated using short-read sequencing on next-generation platforms, this method can sometimes yield low-quality transcripts and incorrect annotations for non-model organisms without a reference genome due to length limitations.^17^ Recently, long-read sequencing technologies have emerged as a solution to these challenges, offering full-length cDNA sequences without the need for assembly. The long-read sequencing application generates full-length cDNA sequences—from the 5ʹ end of transcripts to the poly-A tail—eliminating the need for transcriptome reconstruction using isoform-inference algorithms. It also delivers information about poly-adenylation sites for transcripts up to 10 kb in length across the full complement of isoforms within targeted genes or the entire transcriptome. Extremely long read lengths, eliminating the need for interrupting and splicing, allow direct access to complete information on full-length transcripts that serve as reference genomes for non-model species. This advancement significantly enhances the precision of quantitative gene expression analyses, facilitates the discovery of novel genes and transcriptional isoforms, and enables the identification of variable shear and gene fusion phenomena. Consequently, this technology elevates the accuracy and quality of genetic research endeavours.^18–20^

In this study, we employed PacBio Iso-Seq and RNA-Seq to explore the genome-wide gene expression differences in *L. merzbacheri* populations from two distinct environments: the saline waters of the Jinghe River estuary leading into Ebinur Lake (ABH) and the freshwater of the Boltara River Aheqi Farm (AHQ). We identified gene expression changes against the backdrop of the entire transcriptome. Our research focuses on pinpointing the reactive pathways that respond to high saline stress, utilizing gene ontology and pathway analysis. The insights gained from this study are instrumental in understanding the mechanisms behind the high saline stress tolerance in cyprinidae.

## 2. Materials and methods

### 2.1. Sample collection

we collected six wild *L. merzbacheri* specimens from two distinct environments: the saline waters of the Jinghe River estuary flowing into Ebinur Lake and the fresh waters of the Boltara River Aheqi Farm. We focussed on dissecting and collecting the gill, liver, and kidney tissues due to their significant roles in biotransformation and osmoregulation.^21^ These tissue samples were immediately preserved in liquid nitrogen for subsequent RNA extraction. It is important to note that all animal handling and experimental procedures in this study adhered to the ethical guidelines and received approval from the Animal Care and Use Committee at the Institute of Hydrobiology, Chinese Academy of Sciences.

### 2.2. RNA extraction and quality control

We extracted total RNA from each tissue sample using TRIzol Reagent (Invitrogen, USA), following the manufacturer’s protocol. To remove any potential genomic DNA contamination, the RNA samples were treated with DNase I. The quality and quantity of the RNA were then assessed using gel electrophoresis, a Nanodrop 2000 spectrophotometer (Thermo Fisher Scientific, USA), and an Agilent Bioanalyzer 2100 system (Agilent Technologies, USA). The quality basically meets the requirements of library construction: total quantity ≥ 2 (μg), meeting the requirements of two library construction, concentration ≥ 40 (ng/μl), volume ≥ 10 (μl), OD260/280 between 1.7–2.5, OD260/230 between 0.5–2.5, 260 nm absorption peak display normal. For the PacBio Iso-Seq process, the cDNA library was constructed by mixing 1 μg of total RNA from all tissues of a sample from a AHQ population. Regarding Illumina RNA-Seq, we pooled an equal amount of total RNA from the six fish for each tissue type. Indexed cDNA libraries were then prepared for each tissue sample.

### 2.3. PacBio Iso-Seq library preparation and sequencing

The Iso-Seq library was prepared following the protocol provided by Pacific Biosciences. In summary, we started with 1 μg of total RNA, which was reverse transcribed into full-length cDNA clones (FL-cDNAs) using the Takara SMARTer PCR cDNA Synthesis Kit. We then determined the optimal number of amplification cycles to generate double-stranded DNA (dsDNA). Following amplification, the PCR product was purified using AMPure PB beads (Pacific Biosciences, Menlo Park, CA, USA). This purified product was used for the construction of the SMRTbell library, employing the SMRTBell Template Prep Kit (Pacific Biosciences, Menlo Park, CA, USA). The library was subsequently sequenced on a Pacific Biosciences RS II sequencer, using P2.1-C2.1 chemistry. The sequencing was carried out over a period of 20 h, allowing for extensive data collection (Pacific Biosciences, Menlo Park, CA, USA).

### 2.4. PacBio Iso-Seq sequencing data processing

PacBio Iso-Seq raw data were processed and filtered using the IsoSeq3 v3.8.2 software. Firstly, effective subreads are obtained from raw data with default parameters. Circular consensus sequence (CCS) reads were generated from subread BAM files using pbccs with default parameters. By detecting whether it contains poly(A) signal, 5’ and 3’ primers, CCS sequence is divided into full-length non-chimaera (FLNC) reads and non-full-length (NFL). Finally, full-length reference transcriptome (consensus isoforms) were produced by clustering similar FLNC reads using IsoSeq module with default parameters. Unigenes (high-throughput sequencing identified the gene encoding the protein after multiple contigs were assembled into a scaffold) were obtained by further removing the redundancy through CD-HIT-EST (c = 0.99).^22^

### 2.5. Full-Length transcript functional annotation

The full-length transcripts of *L. merzbacheri* were annotated using the following database: NR (NCBI non-redundant protein sequences), Swiss-Prot (Swiss-Prot Protein Sequence Database), KEGG (Kyoto Encyclopedia of Genes and Genomes), COG (Clusters of Orthologous Groups of proteins), GO (Gene Ontology). The Diamond 2.1.8 software^23^ was employed for functional annotation with an e-value of 1e-10.

### 2.6. Predication of CDS and LncRNA

Identify the potential coding sequences of transcripts and predict the open reading frame (ORF) of transcripts by using TransDecoder 5.7.1 software. Moreover, conducting additional searches and comparisons of the protein sequences of the predicted ORFs with NR and Swiss-Prot using Diamond software can enhance the sensitivity of ORF prediction by providing supporting evidence of homology. The prediction of LncRNA uses RNAsamab 0.2.5^24^ with default parameters.

### 2.7. Illumina RNA-Seq sequencing and data processing

The Illumina library for each tissue sample was constructed using the TruSeq RNA Sample Prep Kit (Illumina, San Diego, CA, USA) following the manufacturer’s instructions. Briefly, the total RNA was fragmented using divalent cations at elevated temperature. The RNA fragments were reverse transcribed into first-strand cDNA using reverse transcriptase and random primers, followed by second-strand cDNA synthesis, end repair, and ligation of the adapters. The ligated fragments were purified and enriched through PCR to generate the final cDNA library. Three samples were sequenced for each tissue of each population, that is, each tissue has three biological repeats. Finally, six transcriptomic libraries were sequenced on Illumina HiSeq X Ten platform to obtain 150 bp pair-end reads. The raw paired-end reads were filtered using fastp 0.23.4^25^ with default parameters.

### 2.8. Read mapping and differential gene expression analysis

All the cleaned reads were mapped to the full-length reference transcriptome by Bowtie2 2.5.1.^26^ RSEM 1.3.3^27^ was then used to estimate and quantify the gene according to the full-length transcriptome. Finally, we used DESeq2^28^ to normalize the expression levels in each of these samples and obtain the differentially expressed transcripts by pairwise comparisons. GO and KEGG pathway enrichment analysis of the differentially expressed genes from the three tissues was performed using the clusterprofiler R package (with the parameter: pvalueCutoff = 0.05).^29^

### 2.9. Quantitative reverse transcription (qRT-PCR)

qRT-PCR was used to validate the RNA-Seq results on randomly nine gene accessions. The beta-actin gene was used as an internal reference, and primers were designed as below, forward primer: 5ʹ-CTTTGAGCAGGAGATGGGAACC-3ʹ; reverse primer: 5ʹ-GATTCCATACCCAGGAAGG-3ʹ. Briefly, qRT-PCR was performed in the optical 96-well plates with an ABI PRISM 7500 Real-time Detection System. The amplification was performed in a total volume of 15 µl, containing 7.5 µl 2X SYBR Green Master Mix reagent, 1 µl of cDNA (100 ng/µl), and 0.3 µl of 10 μM of each gene-specific primer. The PCR cycle was 50°C for 2 min, 95°C for 10 min, 40 cycles of 95°C for 15 s, and 60°C for 1 min. All reactions were set up in triplicate including the negative controls with no template. To assess PCR efficiency, five 10-fold serial dilutions of a randomly selected cDNA sample were used on both the target genes and the reference gene to assess the PCR efficiency. After the PCR, data were analysed with ABI 7500 SDS software. The comparative CT method was used to analyse the expression of the target genes. All data were given at levels relative to the expression of beta-actin gene.

## 3. Results

### 3.1. Full-Length reference transcriptome from PacBio isoform sequencing

**Figure 1.**
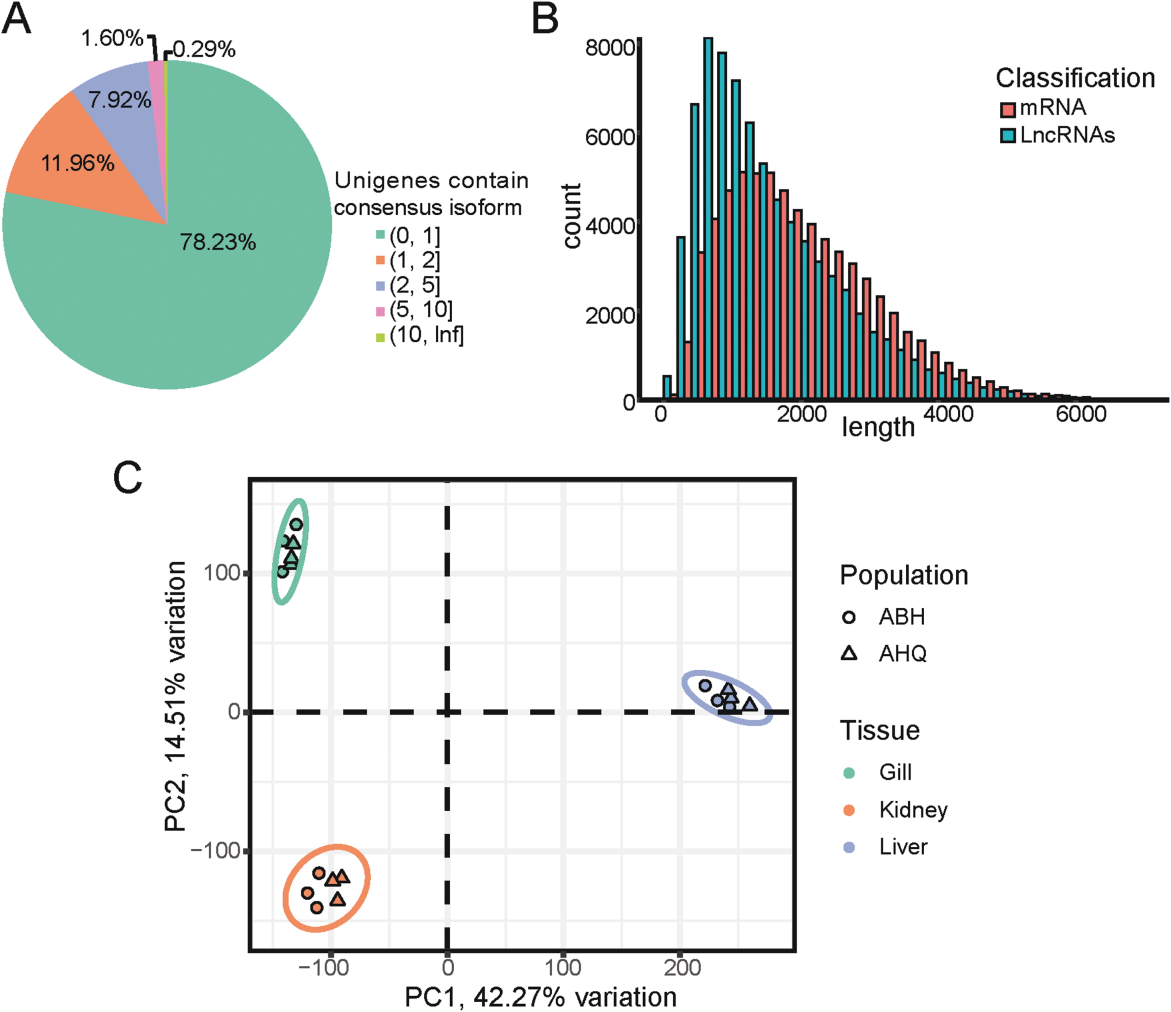
The features of full-length transcripts and exploration of the sample relationships between the two populations of *L. merzbacheri.* (A) Distribution of isoform numbers in unigenes. (B) The length distribution of LncRNA and mRNA transcripts. (C) Principal components 1 and 2 of genes expressed in three tissues of gills, kidneys, and liver of acclimatized ABH and AHQ *L. merzbacheri* populations.

A total of 32,837,398 subreads were obtained from PacBio Iso-Seq, which produced 353,064 CCS reads. CCS reads contained 286,885 FL reads. FL reads comprised 280,929 FLNC reads. Finally, a total of 142,510 full-length transcripts (consensus isoforms) were generated from FLNC reads through collapse. The average length of full-length transcripts was 1,780 bp, and the N50 value was 2.358 bp (Table 1). The unigenes containing 96,861 consensus isoform were obtained after clustering with cd-hit-est (Fig. 1A). 75,771 (78.23%) of the unigenes contain only one consensus isoform, 21,090 (21.77%) contain two or more, 285 (0.29%) contain ten or more and the most containing two is 11,584 (11.96%). The average lenth of unigenes was 1,973 bp, and the N50 value was 2,542 bp.

**Table 1.** Description of the transcroptome of *L. merzbacheri* using PacBio Iso-Seq

Parameters	PacBio Iso-Seq
Number of subreads	32,837,398
Reads of CCS	353,064
Number of FL	286,885
Numer of FLNC	280,929
Full-length transcriptome	-
Number of transcripts	142,510
Average length (bp)	1,780
N50 length (bp)	2,358

### 3.2. Predication LncRNAs and mRNA

The analysis of full-length transcripts led to the prediction of 76,249 lncRNAs based on their protein translation potential. These lncRNAs varied in length, ranging from 50 bp to 9,191 bp, with an average length of 1,516.3 bp. Collectively, they constituted 53.5% of the full-length transcriptome (Fig. 1B). Similarly, 66,262 mRNAs were predicted from the full-length transcripts. The length of these mRNAs ranged from 175 bp to 10,895 bp, with an average of 2,084 bp.

### 3.3. Exploration of sample relationships

The cleaned Illumina reads were mapped onto the full-length transcriptome for gene estimation and quantification. Expression levels in each sample were normalized to delineate sample relationships. Three biological replicates from three different tissues were taken from ABH and AHQ *L. merzbacheri* populations. Principal Component Analysis (PCA) demonstrated distinct tissue-specific clustering of these replicates in the transcriptome data (Fig. 1C). In this dataset, samples grouped according to tissue type across both species, with similar tissues from each species clustering together. The first two principal components (PC1 and PC2) effectively separated the samples by tissue type. This PCA analysis verified the reliability of the sources and the validity of the biological replicates for subsequent analysis. It was observed that similar tissues clustered more distinctly by tissue type rather than by species. This pattern is consistent with findings from previous research by scientists,^30^ suggesting greater variability between tissues than between individuals within a species.

### 3.4. Functional annotation of Full-Length transcriptome

**Figure 2.**
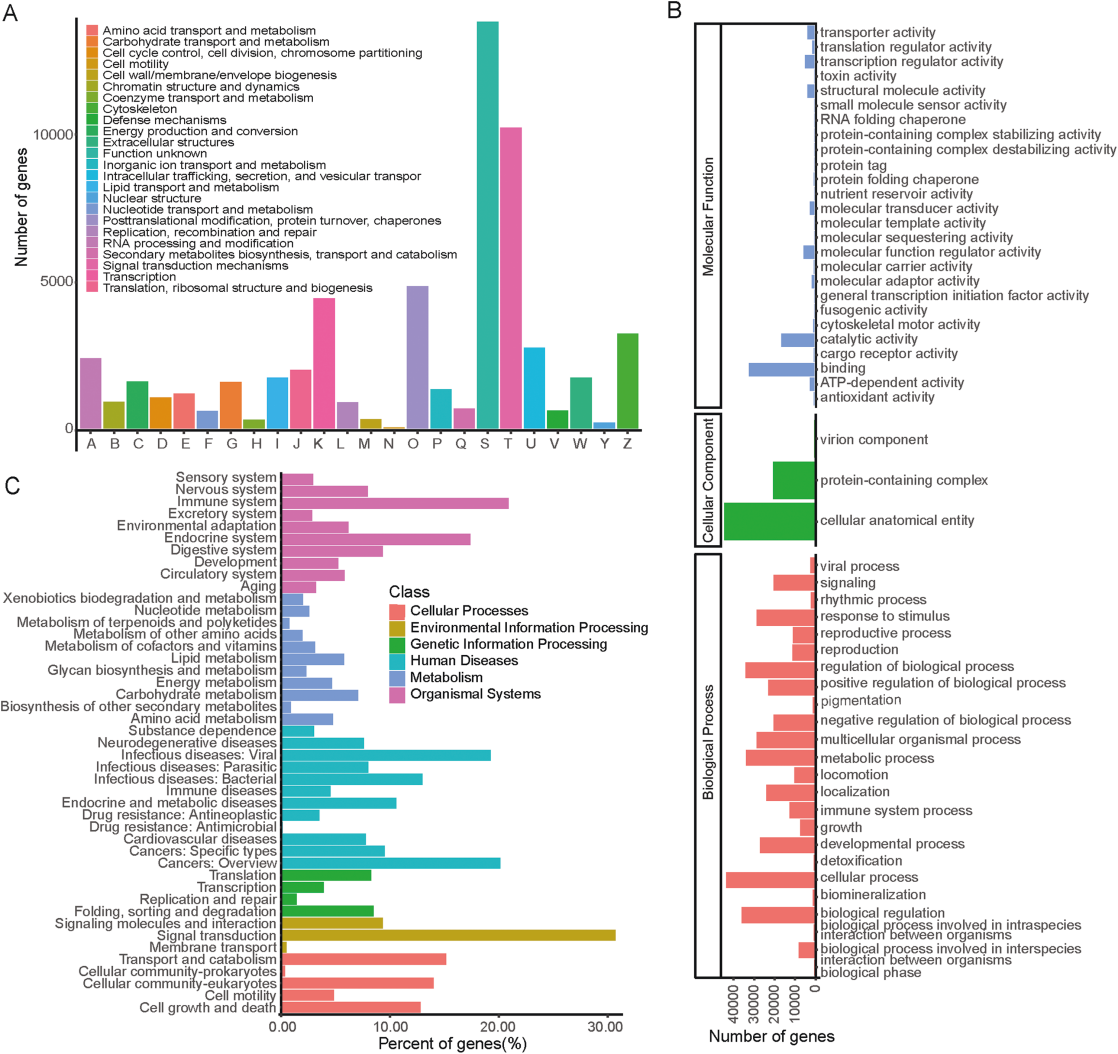
Functional annotation of full-length transcripts from *L. merzbacheri*. (A) COG. (B) GO. (C) KEGG.

The public databases such as EggNOG, GO, COG, and KEGG have been widely used for the functional annotation of full-length transcriptome sequences (Fig. 2). The highest percentage of transcripts was annotated in EggNOG and COG, accounting for 81.82% (54,213) of all transcripts, followed by 68.58% (45,442) in GO and 44.09% (29,217) in KEGG. The number and proportion of full-length transcripts annotated to each public database vary greatly, which may be related to the number of sequences deposited in the databases and their update frequency.^31–33^ GO annotations generated 53 Level 2 GO terms (Fig. 2B). Among them, the four most abundant terms under the biological process category were ‘cellular process’ (43,088), ‘biological regulation’ (35,572), ‘regulation of biological process’ (33,740), and ‘metabolic process’ (33,568). Within the cellular component category, ‘cellular anatomical entity’ (44,042) and ‘protein-containing complex’ (20,033) were the most abundant terms. Of the 26 terms in the molecular function category, ‘binding’ (33,159) and ‘catalytic activity’ (16,718) had the highest number of transcripts. For KEGG annotation, transcripts were mainly assigned to over 437 signalling pathways in 45 Level 2 KEGG groups (Fig. 2C). Among these Level 2 pathways, the signal transduction pathway had the largest number of transcripts (30.66%), followed by the immune system (20.82%), cancers: overview (20.07%), infectious diseases: viral (19.17%), and the endocrine system (17.33%). The COG-annotated transcripts were classified into 24 categories (Fig. 2A), with the highest number of transcripts in ‘function unknown’ (13,746), followed by ‘signal transduction mechanisms’ (10,164), ‘posttranslational modification, protein turnover, chaperones’ (4,805), and ‘transcription’ (4,396).

### 3.5. Differentially expressed genes identified

**Figure 3.**
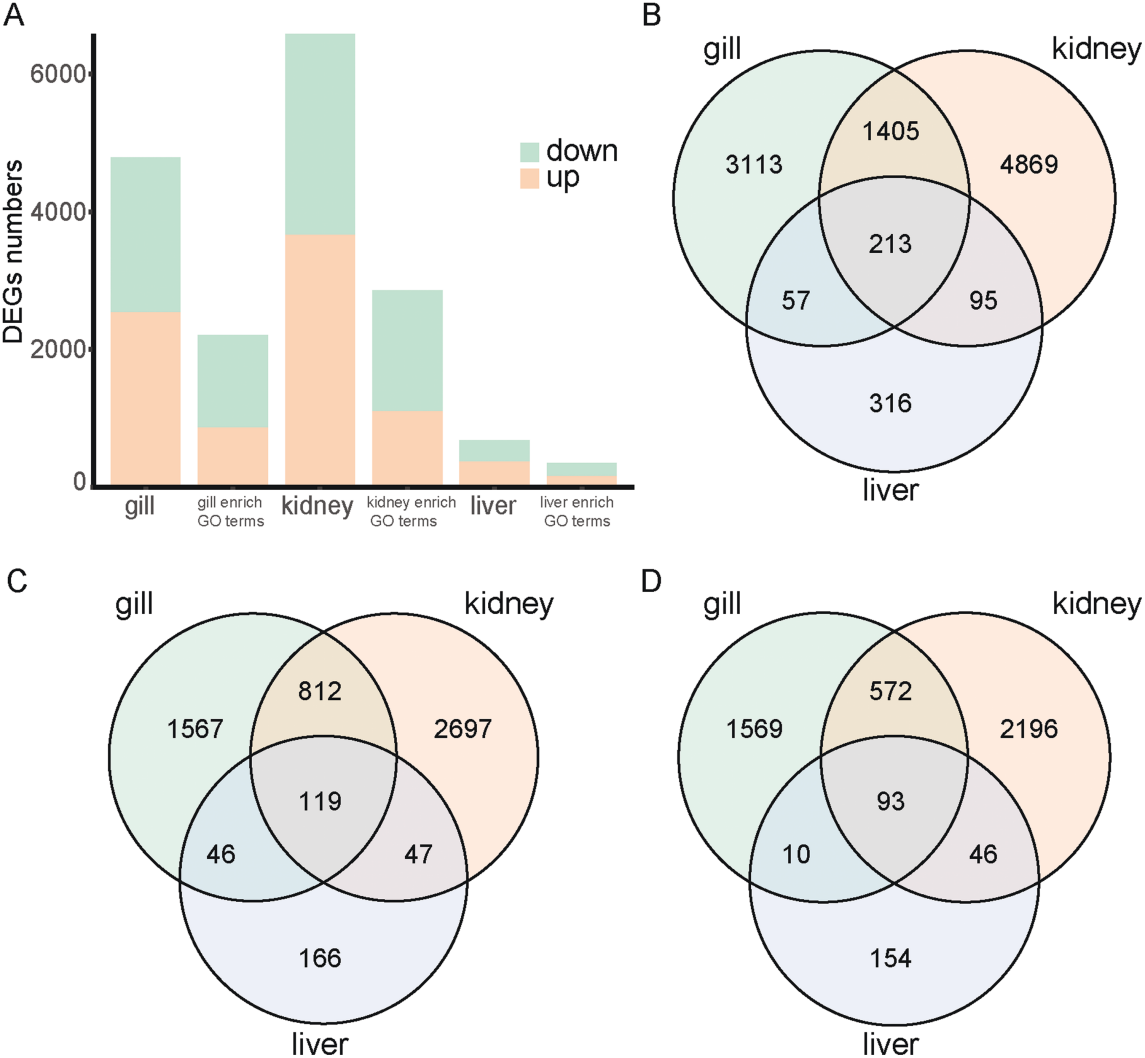
Overview of DEGs across three tissues. (A) Displays the count of DEGs in the three tissues and those enriched in GO terms, where yellow indicates up-regulated DEGs and green signifies down-regulated DEGs. (B) DEGs Veen diagram for three tissues. (C) Veen diagram of up-regulated DEGs in three tissues. (D) Veen diagram of down-regulated DEGs in three tissues.

Utilizing transcript data mapped to the full-length transcriptome, this study aimed to identify differentially expressed genes (DEGs) and explore their potential functions in three tissues of the ABH and AHQ populations. The analysis revealed 681, 6,582, and 4,788 DEGs in the liver, kidney, and gill, respectively, when comparing the ABH population with the AHQ population, with a false discovery rate (FDR) ≤ 0.05 and a log2(fold-change) ≥ 1 (Fig. 3). The top ten DEGs that were up- and down- regulated in each organization are shown in Supplementary Table S1. Of these, 346 in the liver, 2,856 in the kidney, and 2,205 in the gill were enriched for Gene Ontology (GO) terms (Fig. 3A). Specifically, 378, 3,675, and 2,544 genes showed higher expression in the liver, kidney, and gill of the ABH population, respectively, while 303, 2,907, and 2,244 genes demonstrated higher expression in liver, kidney and gill of the AHQ population (Fig. 3). The fish’s adaptation to complex external environments involves significant morphological changes in the gill, kidney, and liver tissues, a critical aspect of establishing adaptive capacity. Differential gene expression correlates with tissue-specific phenotypes and functions. Notably, the number of DEGs varied significantly among the three tissues, with the liver showing a distinct pattern compared to the kidney and gill. The liver exhibited only 681 DEGs, of which 346 were enriched for GO terms. In contrast, the gill and kidney displayed 2,205 and 2,856 GO term-enriched DEGs, respectively. A Venn diagram (Fig. 3) of the DEGs indicated minimal overlap among the three tissues, suggesting distinct mechanistic and pathway responses to saline stress in the liver, kidney, and gill.

**Figure 4.**
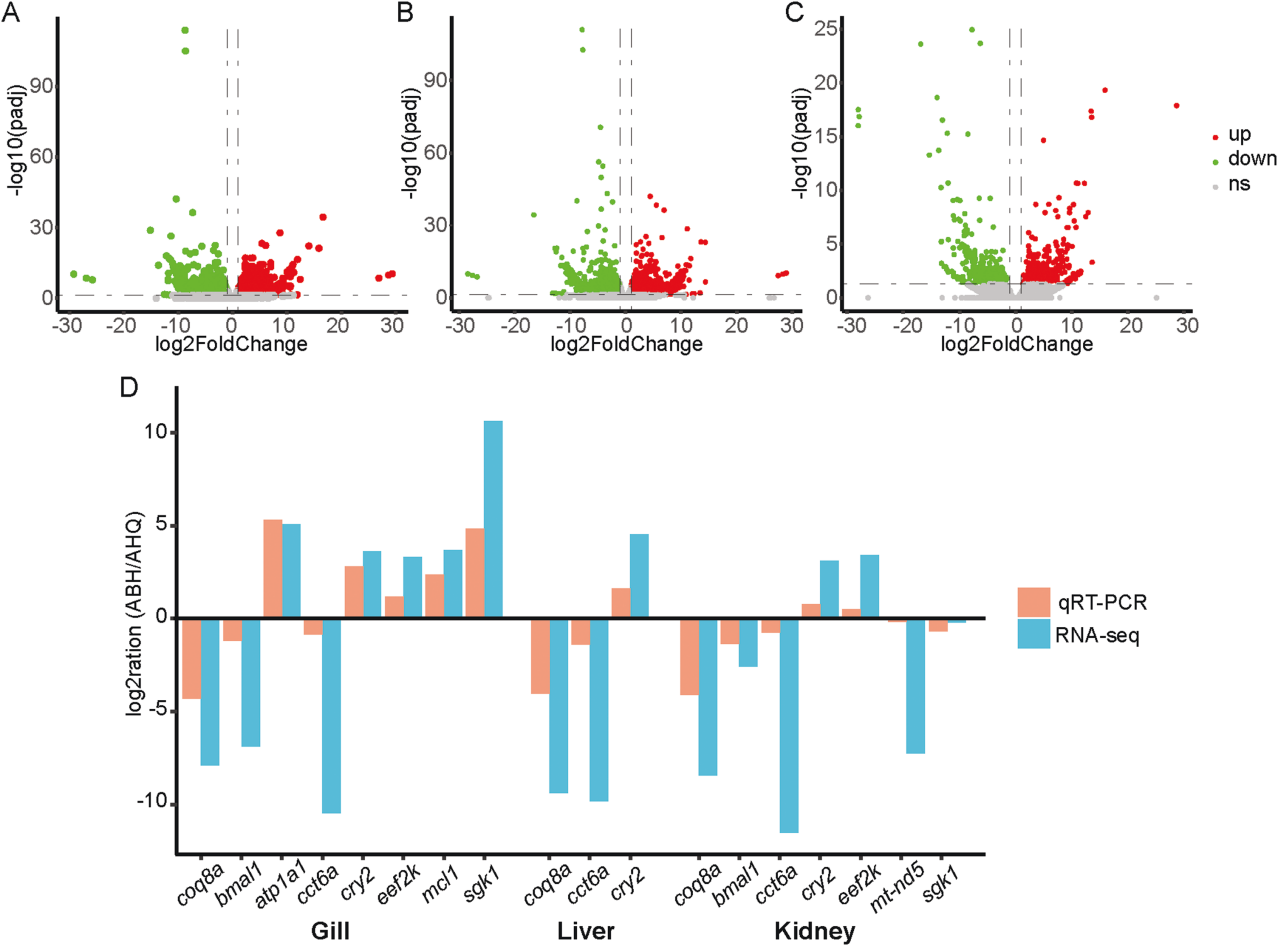
Volcano plots of differentially expressed genes and validation by qRT-PCR. (A) Volcano plots of DEGs in the gill of ABH population to the AHQ population of *L. merzbacheri*. (B) Volcano plots of DEGs in the kidney of ABH population to the AHQ population of *L. merzbacheri*. (C) Volcano plots of DEGs in the liver of ABH population to the AHQ population of *L. merzbacheri*. (D) DEGs validated by qRT-PCR. Comparison between RNA-Seq results and qRT-PCR validation results. X-axis shows genes in three tissues validated in this study; Y-axis shows Log2Ration of expression of ABH versus AHQ.

The volcano plots revealed distinct patterns of differentially expressed genes across three tissues. In the kidney and gill, there was a notable similarity in the down-regulated genes exhibiting highly significant differences in expression (Fig. 4A and B). Meanwhile, the liver showed a more pronounced abundance of both up-regulated and down-regulated genes with significant expression differences (Fig. 4C). However, the subsequent GO enrichment results diverged from those of the volcano plots, possibly because some genes were not annotated to GO terms, or the different genes triggered similar responses within the organism. To corroborate the RNA-Seq findings, we selected 9 genes of considerable importance or with key stress response functions for qRT-PCR analysis, including *serum/glucocorticoid regulated kinase 1* (*sgk1), ATPase Na+/K+ transporting subunit alpha 1 (atp1a1)*, *basic helix-loop-helix ARNT like 1 (bmal1)*, *MCL1 apoptosis regulator, BCL2 family member (mcl1)*, *NADH dehydrogenase 5, mitochondrial (mt-nd5)*, *chaperonin containing TCP1, subunit 6A (cct6a)*, *eukaryotic elongation factor 2 kinase* (*eef2k*), *coenzyme Q8A (coq8a)*, and *cryptochrome circadian regulator 2 (cry2)* for PCR amplification. Among these, the genes *sgk1* and *atp1a1* are involved in regulating ions and osmolality, while the genes *bmal1* and *mcl1* respond to oxidative stress.^34–37^ Genes *mt-nd5* and *coq8a* are integral to the mitochondrial oxidative phosphorylation (OXPHOS) system, while *cct6a* and *eff2k* are associated with protein synthesis.^38^ The gene *cry2* plays a role in regulating endocrine hormones. The number of differentially expressed genes chosen for validation in the gill, liver, and kidney were 8, 3, and 7, respectively. The primers for these genes can be found in Supplementary Table S2. Generally, the gene expression patterns were consistent between the RNA-Seq and qRT-PCR analyses, albeit with variations in expression levels (Fig. 4D). Hence, these genes exhibited similar mRNA expression patterns in both RNA-Seq analysis and qRT-PCR, confirming the genome-wide expression profiling of the liver, kidney, and gill in response to saline stress.

### 3.6. Function analysis on differentially expressed genes in gill

The gill, a crucial organ for maintaining homeostasis, regulates ionic and osmotic pressure, ammonia excretion, gas exchange, and hormone levels in the body.^39^ Among the 4,788 DEGs identified in the gill, we noted a significant up-regulation in genes associated with various GO terms related to endocrine hormones, oxidative stress response, and ion and osmolality regulation. Conversely, down-regulated genes showed significant enrichment in GO terms associated with DNA replication, protein synthesis, and oxidative stress response (Table 2). KEGG pathway enrichment analysis of these DEGs in the gill highlighted significant pathways such as ‘DNA replication’, ‘Renin-angiotensin system’, ‘DNA replication proteins [BR:ko03032]’ and ‘Mismatch repair’. This suggests that DNA and protein synthesis are significantly induced under severe environmental stress in the gills of *L. merzbacheri*.

**Table 2.** GO enrichment analysis of genes that up- or down- regulated in response to saline stress

Tissue	GO term	Definition	Count	Total in category	*p*-Value adjust
Gill	Up				
	GO:0055078	sodium ion homeostasis	16	170	0
	GO:0050886	endocrine process	19	258	0
	GO:0006883	intracellular sodium ion homeostasis	11	100	0
	GO:1902177	positive regulation of oxidative stress-induced intrinsic apoptotic signalling pathway	8	52	0.001
	GO:0060986	endocrine hormone secretion	14	172	0.001
	GO:0036376	sodium ion export across plasma membrane	9	82	0.003
	GO:0044060	regulation of endocrine process	12	165	0.007
	GO:0002028	regulation of sodium ion transport	19	371	0.009
	Down				
	GO:0006261	DNA-templated DNA replication	46	495	0
	GO:0061077	chaperone-mediated protein folding	37	422	0
	GO:0009263	deoxyribonucleotide biosynthetic process	12	54	0
	GO:0042921	glucocorticoid receptor signalling pathway	15	97	0
	GO:0090403	oxidative stress-induced premature senescence	6	14	0
	GO:0009262	deoxyribonucleotide metabolic process	14	101	0
	GO:1903332	regulation of protein folding	16	142	0
	GO:0030174	regulation of DNA-templated DNA replication initiation	7	27	0.001
	GO:0006298	mismatch repair	11	74	0.001
	GO:0034975	protein folding in endoplasmic reticulum	14	123	0.002
	GO:0036500	ATF6-mediated unfolded protein response	11	85	0.004
	GO:0000731	DNA synthesis involved in DNA repair	12	114	0.0129
Kidney	Up				
	GO:0002820	negative regulation of adaptive immune response	17	134	0
	GO:0071454	cellular response to anoxia	6	12	0
	GO:0048102	autophagic cell death	18	181	0.0001
	GO:1900409	positive regulation of cellular response to oxidative stress	15	153	0.0008
	GO:1902884	positive regulation of response to oxidative stress	15	165	0.0018
	GO:1990637	response to prolactin	6	27	0.0034
	GO:0034059	response to anoxia	7	48	0.009
	Down				
	GO:0000963	mitochondrial RNA processing	13	31	0
	GO:0033108	mitochondrial respiratory chain complex assembly	31	201	0
	GO:0010257	NADH dehydrogenase complex assembly	28	168	0
	GO:0032981	mitochondrial respiratory chain complex I assembly	28	168	0
	GO:0061077	chaperone-mediated protein folding	44	422	0
	GO:0006120	mitochondrial electron transport, NADH to ubiquinone	22	136	0
Liver	Up				
	GO:0071454	cellular response to anoxia	5	12	0
	GO:1990637	response to prolactin	5	27	0
	GO:0034059	response to anoxia	5	48	0.0006
	Down				
	GO:0006120	mitochondrial electron transport, NADH to ubiquinone	11	136	0
	GO:0010257	NADH dehydrogenase complex assembly	11	168	0
	GO:0032981	mitochondrial respiratory chain complex I assembly	11	168	0
	GO:0019646	aerobic electron transport chain	13	329	0

### 3.7. Functional analysis on differentially expressed genes in kidney

In response to saline stress in the kidney, our analysis identified a significant enrichment of up-regulated genes across several GO terms. These terms include oxidative stress response, programmed cell death, responses to prolactin and anoxia. These processes have been recognized for their critical roles in stress response and tolerance. Simultaneously, we noted a marked enrichment in down-regulated genes associated with protein synthesis and OXPHOS system (Table 2). Furthermore, KEGG pathway enrichment analysis of these differentially expressed genes in the kidney highlighted a significant presence in the ‘HIF-1 signalling pathway’. Hypoxia-inducible factor-1 (HIF-1) is a transcription factor that orchestrates adaptive responses to oxidative stress, functioning through nuclear translocation and gene expression regulation. It also serves as a key regulator of oxygen homeostasis, emphasizing its central role in cellular adaptation mechanisms.

### 3.8. Function analysis on differentially expressed genes in liver

In the liver’s response to saline stress, our study noted a significant up-regulation of genes associated with several GO terms, notably “response to prolactin and ‘response to anoxia’. Conversely, we observed a substantial enrichment of down-regulated genes in GO terms linked to the OXPHOS system (Table 2). Furthermore, KEGG pathway enrichment analysis of these differentially expressed genes in the liver highlighted a significant presence in the ‘HIF-1 signalling pathway’. The parallelism in GO and KEGG pathway analysis findings between the liver and kidney underscores a similar response mechanism in both organs when dealing with saline stress.

## 4. Discussion

With advancements in sequencing technologies in recent years, long-read sequencing has emerged as a pivotal tool. This technology captures full-length transcripts without the need for further assembly, making it feasible to study the transcriptomes of non-model organisms even in the absence of a reference genome.^40^ In this study, we utilized RNA-seq and PacBio Iso-Seq to report the full-length transcriptome of *L. merzbacheri* for the first time. The average and N50 lengths of these transcripts were 1,780 bp and 2,358 bp, respectively. For functional annotation of the full-length transcriptome sequences, we extensively used public databases such as EggNOG, GO, COG, and KEGG. The majority of transcripts were annotated in EggNOG and COG, comprising 81.82% (54,213) of all transcripts. This was followed by 68.58% (45,442) in GO and 44.09% (29,217) in KEGG. We mapped all the cleaned Illumina reads to the full-length transcriptome for gene estimation and quantification. The expression levels were normalized across each sample to discern sample relationships. This process led to the identification of differentially expressed transcripts by comparing populations in freshwater and saline water environments. In the transcriptome mapping data, samples are grouped by tissue type in both species, with corresponding tissues from each species clustering together. A PCA of all samples revealed that three samples from each tissue type clustered distinctly, confirming the reliability of the source and the efficacy of the biological replicates for subsequent analysis stages. Notably, the same tissues clustered together, displaying more significant differences between tissues than between individuals, aligning with previous scientific findings.^30^ Our analysis revealed 681, 6,582, and 4,788 DEGs in the liver, kidney, and gill, respectively, in the ABH population compared to the AHQ population, with a FDR ≤ 0.05 and log2(fold-change) ≥ 1. Of these, only 346, 2,856, and 2,205 DEGs in the liver, kidney, and gill, respectively, were enriched for GO terms. The Venn diagram (Fig. 3) of the DEGs illustrated that the majority of these genes were unique to each tissue, suggesting that the mechanisms and pathways responding to saline stress significantly differ across the liver, kidney, and gill.

Compared to terrestrial animals, fish require highly efficient mechanisms for regulating ionic and osmotic pressure to maintain acid-base balance in their bodies, a necessity for adapting to the diverse salinities, ionic compositions, and pH levels of aquatic environments.^41^ The gill, kidney, and liver have been identified as crucial organs in developing this adaptive capacity in cyprinidae.

The gill plays a critical role in maintaining homeostasis, regulating ionic and osmotic pressure, facilitating ammonia excretion, gas exchange, and balancing hormone levels in the body.^39^ Our analysis highlighted a significant enrichment of GO terms related to endocrine, sodium ion homeostasis, and transport processes in the up-regulated genes, particularly emphasizing sodium ion export across the plasma membrane. This active sodium ion transport is key to maintaining osmotic pressure homeostasis. This finding suggests that, in response to the hyperosmotic conditions of high saline stress, *L. merzbacheri* actively excretes sodium ions through the gill to preserve osmotic balance. This mechanism of osmotic regulation is similar to that observed in *Alcolapia grahami* and *Alburnus chalcoides* in hypertonic environments.^42–44^ Furthermore, endocrine processes are integral to maintaining bodily homeostasis and regulating metabolism, with endocrine hormones playing a pivotal role in ion transport at various molecular levels.^45^ DNA replication, protein synthesis, and the glucocorticoid receptor signalling pathway were significantly enriched GO terms among the down-regulated genes. DNA replication and protein synthesis are energetically costly and stressed cell usually inhibit this process to redirect resources towards stress response mechanisms. This response is a strategic conservation of energy, enhancing survival during extended periods of stress.^46^ Glucocorticoid receptor binds to cortisol to indirectly regulate sodium ion uptake. Researchers have demonstrated at the molecular level that cortisol binds to the glucocorticoid receptor in zebrafish to mediates sodium-chloride cotransporter (NCC) transcription to regulate sodium ion homeostasis.^47^ Oxidative stress response was significantly enriched in both up- and down-regulated genes, highlighting its strong association with adaptation to saline stress environments.^48^ Notably, the up-regulated genes showed significant enrichment in the positive regulation of oxidative stress-induced intrinsic apoptotic signalling pathway. Apoptosis, a genetically controlled program of cell death, is crucial for maintaining tissue homeostasis.^49^ These results suggest that high levels of programmed cell death may occur in the gills under saline stress, an important insight for understanding the cellular mechanisms of environmental adaptation. KEGG pathway enrichment analysis on those DEGs in gill revealed significant pathway enrichment on rein-angiotensin system (RAS), as well as DNA and protein synthesis. RAS signalling-related pathway were also enriched in treatment experiments with salinity and alkalinity in the *Triplophysa yarkandensis.*^50^ The RAS is a critical system within the body, orchestrating the regulation of water, electrolytes, body fluid balance and blood pressure through a network of proteins, peptides, enzymes, and receptors.^51^ The results of this KEGG pathway enrichment analysis suggest that the RAS, along with DNA and protein synthesis pathways, are significantly activated in response to extreme environmental stress in the gills of *L. merzbacheri.* Drawing from these findings and the GO analysis, we propose that high saline stress impacts both DNA and protein synthesis in the gills, while also intensifying the regulation of sodium ion concentration and osmolality in the gill tissues of the *L. merzbacheri* in the ABH population.

In the kidneys, up-regulated genes were predominantly enriched in GO terms related to oxidative stress response and programmed cell death, similar to the findings in the gills. However, unique to the kidneys, other up-regulated genes were enriched in GO terms associated with responses to prolactin and anoxia. Programmed cell death is a meticulously regulated cellular process that manages cell fate in animals under various cellular stresses or external stimuli. Morphologically, three types of cell death have been identified: type I (apoptosis), type II (autophagic cell death), and type III (necrosis).^52^ The programmed cell death linked to up-regulated gene enrichment in the gills and kidneys corresponds to different types: apoptosis in the gills (type I) and autophagic cell death in the kidneys (type II). Autophagy at physiological levels plays a vital role in maintaining cellular homeostasis under diverse stress conditions. Prolactin (PRL), a key regulator of numerous biological functions in vertebrates, is believed to be crucial for ion uptake in both freshwater and euryhaline fish. It also contributes to reducing ion and water permeability of osmoregulatory surfaces, further emphasizing its significance in the context of fish physiology.^53^ Fish exhibit intricate mechanisms for regulating ionic and osmotic pressure and maintaining acid-base balance to ensure body fluid homeostasis. The endocrine hormone PRL is known to play a pivotal role in this process. It regulates ion transport to maintain osmotic pressure homeostasis by modulating the expression of the ion cotransporter NCC and influencing the differentiation of NCC ionocytes.^45,53–56^ This adaptation involves maintaining high or stable serum ion levels by increasing serum PRL levels, which in turn mediates an increase in Na+/K+-ATPase activity in kidney tissues. This complex interplay of hormonal regulation and enzymatic activity underscores the sophisticated nature of ion homeostasis in fish, particularly in response to varying environmental conditions.^57^ Ammonia (including NH3 and NH4+) is the main metabolic end product of proteins or amino acids that cannot be digested or absorbed in the body and is toxic to all vertebrates.^58^ This ammonia toxicity is a critical factor hindering the adaptation of certain fish species to saline and alkaline environments.^59^ Consequently, efficient and timely ammonia excretion becomes vital for maintaining physiological homeostasis in these animals. Fish that are tolerant of saline and alkaline conditions have developed various strategies to mitigate or circumvent ammonia toxicity, even while enduring higher levels of ammonia in their systems. One effective approach involves lowering their overall metabolic rate to decrease the internal production of endogenous ammonia under saline stress. This adaptation not only diminishes protein oxidation but also ensures that the synthesis of ammonia-N remains below 30%.^60^ Similarly, experiments with *L. waleckii* in Dali Nor Lake under varying alkalinity stresses showed that oxygen consumption rates were substantially lower than those of freshwater counterparts.^61^ This response to anoxia, by reducing oxygen consumption and thereby the overall metabolic rate, is a critical adaptation mechanism that helps minimize or avoid ammonia toxicity, enabling fish to thrive in saline-stressed environments. Down-regulated DEGs in our study were notably enriched in GO terms associated with protein synthesis and the OXPHOS system, both of which are reported to play pivotal roles in stress response and tolerance. The OXPHOS system is a critical pathway for energy production within eukaryotic cells, comprising five main complexes in the electron transport chain: Complex I (NADH: ubiquinone oxidoreductase), Complex II (succinate dehydrogenase), Complex III (cytochrome bc1 oxidoreductase), Complex IV (cytochrome c oxidase), and Complex V (ATP synthase). These complexes work in concert to generate a proton gradient across the mitochondrial membrane, which Complex V then utilizes to synthesize ATP from ADP, fuelling cellular processes.^62^ In both gills and kidneys, down-regulated DEGs showed a significant enrichment in GO terms related to protein synthesis. Given that protein synthesis is an energy-intensive process, cells under stress tend to conserve resources by curtailing this process, redirecting energy towards stress response mechanisms. Our KEGG pathway enrichment analysis of these DEGs in the kidney highlighted a notable enrichment in the ‘HIF-1 signalling pathway’. HIF-1, a complex formed by constitutively expressed β subunits and oxygen-regulated α subunits, plays a crucial role in adapting to oxidative stress.^63^ It achieves this through the nuclear translocation and regulation of gene expression, acting as a fundamental transcription factor in the cellular response to hypoxia.

In the liver, up-regulated DEGs were notably enriched in GO terms related to the response to prolactin and anoxia, while down-regulated DEGs showed significant enrichment in GO terms associated with the OXPHOS system. KEGG pathway analysis further revealed significant enrichment in the HIF-1 signalling pathway among these DEGs. From this analysis, we deduced that the liver’s response to saline stress mirrors that of the kidney closely and to a certain extent, that of the gills as well. The majority of prior investigations into how fish adapt to extreme environments, such as salinity, have predominantly centred on singular tissues or isolated components within tissues.^50,64–66^ Only a limited number of studies have undertaken the analysis of transcriptome alterations across multiple tissues.^67^ In this study, we not only scrutinized the transcriptome changes of three tissues, in response to adaptation to saline environments, but also explored the tissue specificity and relevance of the three tissues to the evolution of salt adaptation. Analysis of the enrichment results across the three tissues reveals a notable similarity between the liver and kidney in terms of their differential gene enrichment pathways. However, the kidney and gill display similarities in pathways associated with oxidative stress response, programmed cell death, and protein synthesis. Intriguingly, there is a distinct lack of significant similarity observed between the liver and kidney in their respective differential gene enrichment pathways. Given that gills serve as the primary tissues exposed to the external environment in fish, while the liver and kidney are situated internally, it follows that DEGs in the gill primarily involve enrichment for sodium ion homeostasis and transport processes. In contrast, the liver and kidney exhibit enrichment for endocrine hormone PRL and response to anoxia. The observed similarity in saline stress adaptation between the gill, kidney, and liver could stem from their parallel functions, challenges in annotating tissue-specific genes with existing GO terms, and the notion that distinct genes may fulfil comparable roles within organisms. Research on salinity tolerance in the obscure pufferfish (*takifugu obscurus)* indicates that osmoregulation in response to salinity involves multiple organs and signalling pathways.^67^ The potential for synergistic mechanisms among them and their interplay warrants further investigation to fully understand the complexity of stress responses in these vital organs.

## Supplementary Material

dsae019_suppl_Supplementary_Table_S1

dsae019_suppl_Supplementary_Table_S2
